# Serum Ceruloplasmin-to-Albumin Ratio as a Biochemical Marker in Pulmonary Tuberculosis Before and After Treatment

**DOI:** 10.7759/cureus.62275

**Published:** 2024-06-12

**Authors:** Rakesh Perumal, Aneesha Konduru, Srinivasan Rengasamy

**Affiliations:** 1 Department of Respiratory Medicine, Meenakshi Academy of Higher Education and Research, Kanchipuram, IND; 2 Department of Respiratory Medicine, Meenakshi Medical College Hospital and Research Institute, Kanchipuram, IND

**Keywords:** serum albumin, serum ceruloplasmin, anti-tuberculosis treatment, serum biomarkers, pulmonary tuberculosis, tuberculosis

## Abstract

Background: The study compares the serum ceruloplasmin-to-albumin ratio of tuberculosis (TB) patients before and after anti-tuberculosis treatment (ATT) to assess its diagnostic and prognostic value. Despite the pandemic's impact on TB notifications, global TB cases rose by 16% in 2022.

Methods: The study was conducted at Meenakshi Medical College Hospital and Research Institute, Kanchipuram, from November 2022 to November 2023, with participants aged 15 and above diagnosed with pulmonary TB. The analysis of clinical, radiographic, microbiological, and biochemical data revealed a gender distribution of 58% male and 42% female individuals, with an average age of 49. Significant reductions in ceruloplasmin levels and increases in albumin levels were found following therapy, as well as a decrease in the ceruloplasmin-to-albumin ratio, showing that ceruloplasmin may serve as a severity measure and treatment indicator.

Results: Male patients accounted for 58% of the study population, while females accounted for 42%. Patients aged 36-45 made up the largest group (26%). Following treatment, serum ceruloplasmin levels decreased significantly (from 66.28 mg/dL to 35.56 mg/dL), but albumin levels increased (from 2.96 g/dL to 4.19 g/dL). The ceruloplasmin-to-albumin ratio dropped from 0.022 to 0.008, showing treatment efficacy.

Conclusions: The study highlights the potential of serum biomarkers for diagnosing and monitoring TB. The serum ceruloplasmin-to-albumin ratio is a promising biochemical diagnostic. Further research is needed to validate these findings and investigate their clinical significance in TB management.

## Introduction

Tuberculosis (TB) continues to be a formidable global health challenge, ranking among the primary infectious diseases contributing to mortality worldwide. Despite strides in diagnosis, treatment, and prevention, TB persists as a significant public health burden, particularly in low- and middle-income nations. The clinical presentation of TB exhibits considerable diversity among individuals, influenced by factors such as the infecting dose's characteristics, environmental conditions supporting the persistence of infectious particles, and the virulence of the bacterial strain. Despite variations in individual susceptibility, those afflicted face a lifetime risk of developing active TB, estimated between 5% and 10% [1].

The global incidence of newly diagnosed TB cases has undergone marked fluctuations due to the COVID-19 pandemic's impact on TB case notifications. Following notable escalations from 2017 to 2019, reported TB cases declined by 18% from 2019 to 2020, decreasing from 7.1 million to 5.8 million cases. This reduction was attributed to pandemic-related disruptions in healthcare services, impeding TB diagnosis and reporting. However, there was a slight rebound to 6.4 million cases in 2021 as healthcare systems began recovery. Remarkably, in 2022, the number of reported TB cases surged to pre-pandemic levels, with 7.5 million new diagnoses. This 16% increase compared to 2021 suggests improved healthcare service availability and potentially indicates the identification of a backlog of TB cases and an uptick in new TB infections [1,2].

Significantly, the WHO African Region experienced increased TB case notifications throughout the pandemic, indicating minimal disruptions in TB detection due to COVID-19 [3]. Conversely, other regions encountered substantial challenges in maintaining TB diagnostic services, leading to delayed diagnoses and treatment interruptions. At the national level, reported TB cases exhibited significant variability during and after the pandemic, categorizing countries into six groups based on the extent of disruptions and recovery patterns.

In light of these challenges, it is imperative to enhance monitoring and response measures to mitigate TB's impact, particularly in the post-pandemic period. Understanding the dynamics of TB case notifications amid the COVID-19 pandemic is crucial for formulating targeted interventions and optimizing strategies for TB management in the future. Enhanced surveillance systems, improved diagnostic capabilities, and sustained public health efforts are essential to address the resurgence of TB and prevent further setbacks in global TB control efforts [4].

There is a growing need for reliable biomarkers to aid in TB diagnosis, prognosis, and monitoring. The serum ceruloplasmin-to-albumin ratio has emerged as a potential candidate in this regard. Ceruloplasmin, an acute-phase reactant with ferroxidase activity, and albumin, a negative acute-phase protein, may reflect the host's response to TB infection through changes in their levels during infection and inflammation. The present study aims to assess the serum ceruloplasmin-to-albumin ratio in patients with pulmonary tuberculosis (PTB), investigating its potential as a diagnostic and prognostic marker in TB therapy. Additionally, the study seeks to compare the serum ceruloplasmin-to-albumin ratio in patients with PTB before and after undergoing anti-tuberculosis treatment (ATT) [5]. This investigation will explore the utility of these ratios in monitoring treatment response and disease progression.

## Materials and methods

Study design and patient selection

The study was conducted at the respiratory outpatient and inpatient department of the Department of Respiratory Medicine, Meenakshi Medical College Hospital and Research Institute, Kanchipuram, from November 2022 to November 2023. Utilizing a cross-sectional design, the study focused on individuals newly diagnosed with microbiologically confirmed PTB (50 cases) aged over 15, encompassing both male and female patients. Subsequently, these patients were monitored after completing their ATT.

Data collection and analysis

Data collected during the study period were inputted into Microsoft Excel and subsequently analyzed using IBM SPSS Statistics for Windows, V. 25.0 (IBM Corp., Armonk, NY) for statistical analysis [6]. Inclusion criteria targeted newly diagnosed patients with microbiologically confirmed PTB aged over 15, without gender restrictions. In contrast, exclusion criteria included patients with extrapulmonary TB and multidrug-resistant TB, as well as those with pulmonary infections unrelated to TB, with renal or liver problems, who are pregnant or breastfeeding, with human immunodeficiency virus (HIV) infection, and with malignancies such as leukemia, lymphoma, or breast cancer. These criteria ensured a homogeneous study population and minimized potential confounding factors influencing the outcomes.

Clinical evaluation and diagnostic procedures

Upon enrollment, patients underwent a comprehensive medical history assessment focusing on symptoms indicative of PTB, such as fever, weight loss, reduced appetite, and a persistent cough with phlegm for over two weeks. Comprehensive evaluations were conducted, encompassing clinical, radiographic, microbiological, and biochemical investigations. Laboratory tests included complete blood counts, erythrocyte sedimentation rates, renal function tests, liver function tests, random blood sugar levels, HIV tests, and electrocardiograms (ECGs). Chest X-rays were obtained, and sputum samples were collected for acid-fast bacilli (AFB) staining and GeneXpert analysis. Two sputum samples, one in a fasting state and the other postprandial, were collected, known as early morning and spot samples, respectively. Auramine-rhodamine staining was employed to identify AFB in sputum samples. Patients testing positive for *Mycobacterium tuberculosis* (MTB) via AFB and GeneXpert tests commenced ATT [7].

TB treatment protocol

Patients received a six-month regimen of anti-TB medication, comprising an initial intensive phase followed by a continuation phase. The initial phase involved administering isoniazid, rifampicin, pyrazinamide, and ethambutol, while the continuation phase included isoniazid, rifampicin, and ethambutol. Medication dosages were adjusted based on patient weight, with isoniazid administered at 5 mg/kg, rifampicin at 10 mg/kg, ethambutol at 15 mg/kg, and pyrazinamide at 25 mg/kg. Prior to treatment initiation, serum ceruloplasmin and serum albumin levels were measured. Throughout treatment, regular chest X-rays and sputum AFB examinations were conducted to monitor progress and detect potential adverse effects. Post-treatment, serum ceruloplasmin and albumin levels were re-evaluated [5].

Auramine-rhodamine stain

The detection of MTB bacilli using the fluorescent staining method with auramine-rhodamine stain is essential in TB diagnosis. This protocol involves preparing and fixing sputum specimens, staining with auramine-rhodamine solution, decolorizing, and counterstaining. Stained smears are examined under a fluorescence microscope for the characteristic fluorescence pattern of AFB. This method provides the rapid detection of MTB bacilli with high sensitivity and is widely utilized in clinical laboratories for diagnosing PTB [8].

Bromocresol green (BCG)-induced serum albumin

The determination of serum albumin using the BCG method involves a photometric assay performed under mildly acidic conditions. The interaction between BCG and serum albumin results in a color change from yellowish-green to bluish-green. This technique uses citrate buffer and BCG at concentrations of 30 mmol/L and 0.26 mmol/L, respectively. The linear range for measuring serum albumin is up to 6 g/dL. If a sample exceeds this range, it is diluted with a NaCl solution (9 g/L) in a 1:1 ratio, and the resulting value is multiplied by 2. The normal range for albumin levels is 3.5-5.2 g/dL [8].

Measurement of serum ceruloplasmin via immunoturbidometry

Immunoturbidometry is employed to estimate serum ceruloplasmin levels. This technique is based on an immunoturbidometric reaction between polyclonal antiserum against ceruloplasmin and its corresponding antigen. This reaction occurs under optimal conditions, such as the appropriate pH and the presence of polyethylene glycol, leading to the formation of turbidity proportional to the ceruloplasmin concentration. Essential reagents include concentrated phosphate buffer, polyethylene glycol, and anti-ceruloplasmin polyclonal antiserum. The normal range for serum ceruloplasmin in adults is 20-60 mg/dL [8].

## Results

Distribution of PTB patients by gender

Our study included evaluations of 29 male patients and 21 female patients, both before and after ATT. The study population consisted of 58% males and 42% females (Table 1). The mean age (standard deviation) of the female population was 53 years ± 11.65, while for the male population, it was 42 years ± 13.24. Among male participants, the youngest patient was 32 years old, while the oldest patient was 70 years old. For female participants, the youngest patient was 23 years old, and the oldest was 65 years old. This indicates a notable variation in age distribution between male and female participants in our study.

**Table 1:** Gender-wise distribution of PTB patients PTB: pulmonary tuberculosis

Gender distribution	Number of patients	Percentage
Male	29	58%
Female	21	42%
Total	50	100%

Age distribution of study participants

In our study, the mean age of our patients was 49 ± 13.37 years. The age range among our study population varied from 23 to 70 years. Among the different age groups, the highest proportion of patients, constituting 26% of our study population, fell within the age range of 36-45 years. Patients aged between 46 and 55 years, as well as those aged between 56 and 65 years, each comprised 22% of the total population. Patients in the age groups of 26-35 years and above 65 years constituted 12% each. Lastly, patients below the age of 25 years represented 6% of the total population (Table 2).

**Table 2:** Age-wise distribution of PTB The mean age of the study participants was 49.18 years ± 13.37. Age-wise distribution revealed varying proportions across different age brackets, with a significant representation observed in the 36-45-year age group, accounting for 26% of the total population. PTB: pulmonary tuberculosis

Age distribution	Number of patients	Percentage
≤25 yrs	3	6%
26-35 yrs	6	12%
36-45 yrs	13	26%
46-55 yrs	11	22%
56-65 yrs	11	22%
>65 yrs	6	12%
Total	50	100%

Comparison and estimation of serum ceruloplasmin and serum albumin values before and after ATT

In our study, we compared the mean values of serum ceruloplasmin before and after ATT. Before treatment, the mean value of serum ceruloplasmin was 66.28 mg/dL, whereas after treatment, it decreased to 35.56 mg/dL. This difference was statistically significant (t-value = 22.015, p-value <0.0001), indicating a substantial reduction in serum ceruloplasmin levels following ATT (Table 3).

**Table 3:** Comparison of serum ceruloplasmin and albumin values in PTB patients before and after ATT The table presents mean values, standard deviations, and minimum and maximum values, along with t-values and corresponding p-values for statistical analysis. PTB: pulmonary tuberculosis; ATT: anti-tuberculosis treatment

Variable	Group	N	Mean ± SD	Min	Max	T-value	P-value
Serum ceruloplasmin	Before ATT	50	66.28 ± 7.69	51.00	82.00	22.015	0.0001
	After ATT	50	35.56 ± 6.17	28.00	55.00		
Serum albumin	Before ATT	50	2.96 ± 0.21	2.50	3.30	-22.187	0.0001
	After ATT	50	4.19 ± 0.33	3.60	5.10		
Serum ceruloplasmin-to-albumin ratio	Before ATT	50	0.022 ± 0.003	0.02	0.03	27.661	0.0001
	After ATT	50	0.008 ± 0.001	0.01	0.01		

We analyzed the mean values of serum albumin before and after ATT in PTB patients. Before treatment, the mean serum albumin level was 2.96 g/dL, with a standard deviation of ±0.21, while after treatment, it increased to 4.19 g/dL, with a standard deviation of ±0.33. This increase in serum albumin levels after ATT was statistically significant (t-value = -22.187, p-value <0.0001), indicating an improvement in albumin levels post-treatment. The table summarizes the comparison of serum albumin values before and after ATT, indicating a significant increase in albumin levels post-treatment.

## Discussion

PTB posed a substantial public health challenge in many nations, including India. The disease, caused by MTB, is notorious for its high infectivity and potential to induce severe health complications. According to the Global TB Report 2021, over 3.6 million cases were categorized as "missing," indicating either a lack of diagnosis or underreporting to national TB programs. India alone accounted for one-third of these cases and constituted a quarter of global incident cases [1-2]. Prompt and precise diagnosis followed by timely treatment is pivotal in interrupting TB transmission.

Presently, diagnosing MTB relies on a blend of microbiological and radiological assessments. Mycobacterial culture, whether solid or liquid, remains the gold standard due to its high sensitivity [3]. Nonetheless, the culture process is time-intensive, taking a minimum of two to three weeks for results and up to eight to 12 weeks to confirm a negative outcome [4]. This prolonged duration poses a significant hurdle in promptly treating PTB.

For suspected PTB cases, initial diagnostic steps involve sputum assessment via smear microscopy and molecular techniques to screen for drug resistance. Simultaneously, a chest X-ray is conducted, highly sensitive in detecting anomalies but less specific in TB diagnosis [5]. Rapid screening techniques like the AFB smear are beneficial yet not always effective, particularly in cases with low bacterial loads. Serum biomarkers have emerged as valuable tools in diagnosing PTB, especially in patients with symptoms but negative sputum outcomes. Our investigation focused on serum biomarkers ceruloplasmin and albumin in patients pre- and post-ATT [6-7].

In our study, conducted from November 2022 to November 2023 at the Meenakshi Medical College Hospital and Research Institute, male patients comprised 58% of the cohort, with females accounting for 42%. Results suggested a slightly higher propensity for PTB among males compared to females. Most patients fell within the 35-45 age bracket, constituting 26% of the population. The age range spanned from 23 to 70 years, with 88% aged between 26 and 65 years. Notably, female patients were diagnosed at a mean age of 42 years, approximately 10 years younger than male patients (mean age: 53 years) [8].

Ceruloplasmin, an acute-phase reactant, increases in response to inflammation and infection. In our study, mean serum ceruloplasmin levels in newly diagnosed PTB patients were 66.28 ± 7.69 mg/dL, decreasing to 35.56 ± 6.17 mg/dL post-treatment. This significant reduction suggests a resolution of inflammation and infection through effective treatment [9-10]. Conversely, albumin, a negative acute-phase reactant, decreases in chronic inflammation due to increased vascular permeability and albumin leakage into tissues. In our study, mean serum albumin levels in newly diagnosed TB patients were 2.96 ± 0.21 g/dL, rising to 4.19 ± 0.33 g/dL post-treatment, indicating recovery from TB-induced inflammation [11-12].

The serum ceruloplasmin-to-albumin ratio in newly diagnosed TB patients was 0.022 ± 0.003, decreasing to 0.008 ± 0.001 post-treatment. This decline supports the utility of these biomarkers in assessing disease severity and treatment response [13-15]. Our research underscores the potential of serum biomarkers not only in diagnosing PTB but also in monitoring treatment efficacy. Our findings highlight significant changes in serum ceruloplasmin and albumin levels pre- and post-ATT, supporting their role as biomarkers in TB management. Further investigation is warranted to elucidate underlying mechanisms and validate the practical application of these biomarkers in larger and more diverse patient cohorts.

Limitations

Despite its contributions, this study is not without limitations. Firstly, the relatively small sample size of 50 patients may limit the generalizability of findings and statistical power. Secondly, the absence of a control group for comparison could hinder the interpretation of observed changes in serum biomarkers. Additionally, the study's short follow-up period may overlook the potential long-term effects of ATT on serum biomarkers and disease outcomes. Factors such as diet, comorbidities, and medication adherence were not thoroughly assessed or controlled for, potentially confounding observed associations. Being a single-center study, the lack of diversity in patient populations and clinical settings may limit the study's external validity. Lastly, while significant changes in serum biomarkers were observed, the specific clinical implications for diagnosis and treatment outcomes remain unclear and warrant further investigation.

## Conclusions

We investigated the levels of serum ceruloplasmin and albumin and the ceruloplasmin-to-albumin ratio in patients with PTB both before and after receiving ATT. The findings of our study suggest that untreated patients had higher amounts of ceruloplasmin in their blood serum and lower levels of albumin. However, these levels return to normal after therapy. Furthermore, TB patients exhibited a greater ceruloplasmin-to-albumin ratio, which subsequently decreased following the end of treatment. The data indicate that the serum ceruloplasmin-to-albumin ratio has the potential to be used as a biochemical marker in PTB. Additional investigation is necessary to validate these findings and investigate their clinical importance in the management of TB.

## References

[1] (2024). Global tuberculosis report 2023. https://www.who.int/teams/global-tuberculosis-programme/tb-reports/global-tuberculosis-report-2023.

[2] Cai Y, Dai Y, Wang Y (2020). Single-cell transcriptomics of blood reveals a natural killer cell subset depletion in tuberculosis. EBioMedicine.

[3] Morrison H, McShane H (2021). Local pulmonary immunological biomarkers in tuberculosis. Front Immunol.

[4] Li H, Zhou Q, Ding Z, Wang Q (2023). RTP4, a biomarker associated with diagnosing pulmonary tuberculosis and pan-cancer analysis. Mediators Inflamm.

[5] Shaukat SN, Eugenin E, Nasir F, Khanani R, Kazmi SU (2023). Identification of immune biomarkers in recent active pulmonary tuberculosis. Sci Rep.

[6] (2024). Downloading IBM SPSS Statistics 29.0.2.0. https://www.ibm.com/support/pages/downloading-ibm-spss-statistics-29020.

[7] Hareedy MS, Tawfik KM (2022). Systemic isotretinoin has an impact on hemoglobin, ferritin, urea, ceruloplasmin, albumin, uric acid levels, and neutrophil to lymphocyte ratio in acne patients. J Cosmet Dermatol.

[8] Cattaneo L, Lopreiato V, Piccioli-Cappelli F, Trevisi E, Minuti A (2021). Plasma albumin-to-globulin ratio before dry-off as a possible index of inflammatory status and performance in the subsequent lactation in dairy cows. J Dairy Sci.

[9] Razavi F, Sheikholeslami S, Zarif Yeganeh M, Naji T, Hedayati M (2020). Ceruloplasmin status in medullary thyroid carcinoma. Iran J Public Health.

[10] Muraviov AN, Vinogradova TI, Remezova AN (2022). The use of mesenchymal stem cells in the complex treatment of kidney tuberculosis (experimental study). Biomedicines.

[11] Kaushik SR, Sahu S, Guha H (2022). Low circulatory Fe and Se levels with a higher IL-6/IL-10 ratio provide nutritional immunity in tuberculosis. Front Immunol.

[12] Kinkar E, Kinkar A, Saleh M (2019). The multicopper oxidase of Mycobacterium tuberculosis (MmcO) exhibits ferroxidase activity and scavenges reactive oxygen species in activated THP-1 cells. Int J Med Microbiol.

[13] Parida A, Mohanty A, Raut RK, Padhy I, Behera RK (2023). Modification of 4-fold and B-pores in bacterioferritin from Mycobacterium tuberculosis reveals their role in Fe2+ entry and oxidoreductase activity. Inorg Chem.

[14] Lee CY, Hong JY, Lee MG, Suh IB (2017). Identification of 10 candidate biomarkers distinguishing tuberculous and malignant pleural fluid by proteomic methods. Yonsei Med J.

[15] Janciauskiene S, Royer PJ, Fuge J (2020). Plasma acute phase proteins as predictors of chronic lung allograft dysfunction in lung transplant recipients. J Inflamm Res.

